# Neoadjuvant immunotherapy for resectable esophageal cancer: A review

**DOI:** 10.3389/fimmu.2022.1051841

**Published:** 2022-12-08

**Authors:** Qing Li, Ting Liu, Zhenyu Ding

**Affiliations:** Department of Biotherapy, Cancer Center, West China Hospital, Sichuan University, Chengdu, China

**Keywords:** esophageal cancer (EC), immune checkpoint inhibitor (ICI), immunotherapy, neoadjuvant therapy, chemotherapy, radiotherapy

## Abstract

Esophageal cancer (EC) is one of the most common cancers worldwide, especially in China. Despite therapeutic advances, the 5-year survival rate of EC is still dismal. For patients with resectable disease, neoadjuvant chemoradiotherapy (nCRT) in combination with esophagectomy is the mainstay of treatment. However, the pathological complete response (pCR) rate to nCRT of 29.2% to 43.2% is not satisfactory, and approximately half of the patients will develop either a locoregional recurrence or distant metastasis. It is, therefore, necessary to explore novel and effective treatment strategies to improve the clinical efficacy of treatment. Immunotherapy utilizing immune checkpoint inhibitors (ICIs) has significantly changed the treatment paradigm for a wide variety of advanced cancers, including EC. More recently, increasing clinical evidence has demonstrated that neoadjuvant immunotherapy can potentially improve the survival of patients with resectable cancers. Furthermore, accumulating findings support the idea that chemotherapy and/or radiotherapy can activate the immune system through a variety of mechanisms, so a combination of chemotherapy and/or radiotherapy with immunotherapy can have a synergistic antitumor effect. Therefore, it is reasonable to evaluate the role of neoadjuvant immunotherapy for patients with surgically resectable EC. In this review, we discuss the rationale for neoadjuvant immunotherapy in patients with EC, summarize the current results of utilizing this strategy, review the planned and ongoing studies, and highlight the challenges and future research needs.

## Introduction

Esophageal cancer (EC) is the sixth leading cause of cancer-related mortality worldwide, with approximately 544,000 deaths from EC in 2020 (1). In contrast to Western countries, esophageal squamous cell carcinoma (ESCC) accounts for approximately 90% of EC cases in East Asia (1, 2). Surgery remains the mainstay for the treatment of early-stage EC. However, most patients with EC are already in a locally advanced stage at the time of diagnosis, and surgery alone has a limited effect, with a 5-year survival rate of only 25% (3). For resectable locally advanced EC, neoadjuvant chemoradiotherapy (nCRT) could improve survival compared to surgery alone (4, 5). Therefore, preoperative nCRT followed by surgery has become the standard of care for these patients (6). However, nearly half of patients still develop local recurrence or distant metastases after surgery (4). It is therefore necessary to explore novel and effective treatments to improve survival.

In recent years, immune checkpoint inhibitors (ICIs) have made significant advances in a variety of tumors (7, 8). In EC, the KEYNOTE-181 study showed that compared with chemotherapy, pembrolizumab demonstrated a longer overall survival (OS, 6.7 vs. 9.3 months), a higher objective response rate (ORR, 7.4% vs. 16.7%) and a lower incidence of grade 3-5 adverse events (AEs, 40.9% vs. 18.2%) as 2nd-line treatment (9). In addition, the RATIONALE-302 (10), ATTRACTION-3 (11) and ESCORT studies (12) all showed positive results in similar populations. The latest results from the JUPITER-06 (13), CheckMate 648 (14), ORIENT-15 (15), ESCORT-1st (16) and KEYNOTE-590 (17) studies showed that treatment of patients with advanced EC with programmed death 1 (PD-1) inhibitors plus chemotherapy as 1^st^-line therapy resulted in significantly longer OS and progression-free survival (PFS) than chemotherapy alone. These results suggest that ICIs have promising prospects for EC treatment.

Currently, ICI neoadjuvant therapy has been tried in a variety of tumors, such as lung cancer (18, 19), melanoma (20–23), bladder cancer (24), colon cancer (25) and glioblastoma (26, 27). ICI neoadjuvant therapy for EC is also being actively explored. In this review, we will describe the rationale for ICI neoadjuvant therapy in EC, the reported outcomes, the planned and ongoing studies, the unresolved issues, and the directions for future research.

## Rationale of neoadjuvant therapy

### Biological basis of EC

Antitumor immune responses can be driven by mutation-associated neoantigens that are recognized as nonself-foreigners by T cells that have escaped negative selection during T-cell development (28). Tumor mutational burden (TMB) is a prototype measure of tumor foreignness that reflects the diversity of neoantigens (28). Therefore, a high TMB is positively correlated with the efficacy of ICIs (29–32), and the US Food and Drug Administration approved TMB as a companion diagnostic biomarker as an indication for using the PD-1 inhibitor pembrolizumab to treat patients with unresectable or metastatic solid tumors. The genomic aberrations in EC have been comprehensively studied (33–38), and a high TMB occurs in most cases of EC (39, 40). In addition, programmed cell death-ligand 1 (PD-L1) is widely expressed in EC cells and is associated with a poor prognosis (41–43). In a pooled analysis, PD-L1 overexpression was found in 559/1,350 ESCC patients (41.4%) (42). For patients with ESCC, PD-L1 was negatively associated with a pathological complete response (pCR, 13% vs. 32%) after nCRT treatment (44, 45). Furthermore, PD-L1 expression also predicts a high postoperative recurrence rate and low survival rate in ESCC patients (46). Not surprisingly, anti-PD-1 antibodies show good clinical efficacy and safety for the treatment of advanced EC (9–17). It is also reasonable to evaluate the role of ICIs in preoperative treatment.

### Actions of ICIs

**Figure 1:**
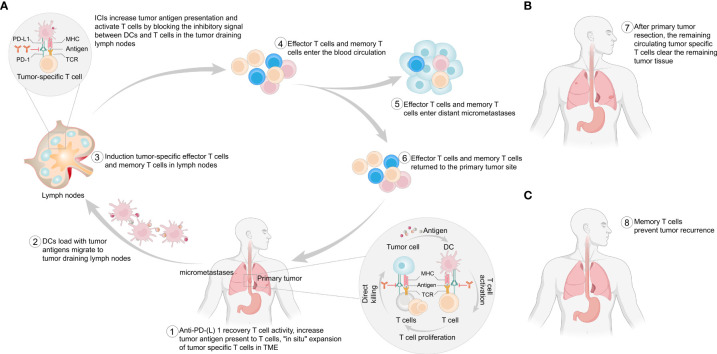
Potential mechanism of neoadjuvant immunotherapy. **(A)** This figure provides a stepwise overview of the potential mechanism of the antitumor effect of ICIs in the presence of a primary tumor. **(B)** After surgical removal of the primary tumor, T cells in the blood circulation can continue to exert antitumor effects to clear any remaining tumor cells. **(C)** After surgical removal of the primary tumor, immune memory cells prevent any postoperative recurrence and metastasis. PD-1, programmed cell death protein 1; PD-L1, programmed cell death 1 ligand 1; MHC, major histocompatibility complex; TCR, T-cell receptor; TME, tumor microenvironment; DCs, dendritic cells.

Anti-PD-(L)1 antibodies block the inhibitory signals between tumor cells and T cells in the tumor microenvironment (TME), reversing the exhausted state of T cells (47–49). Dendritic cells (DCs) originating from primary tumors take up tumor antigens and traffic to tumor-draining lymph nodes, where they present antigens in an ineffective or tolerogenic manner to tumor-specific T cells. Anti-PD-(L)1 antibodies also increase antigen presentation by blocking the inhibitory signals between PD-L1-expressing DCs and T cells, resulting in the “in situ” expansion of tumor-specific T cells. These activated T cells enter the blood circulation or lymphatic vessels and then enter the primary tumor tissue or distant micrometastases to exert antitumor effects. The presence of a primary tumor allows the induction of a broader and stronger T-cell response (48, 49) (**Figure 1**). In addition, tumor-specific T cells in the blood circulation continue to clear residual tumor cells after surgery (49) (**Figure 1**). Moreover, preoperative immunotherapy can activate the patient’s immune system to form immune memory cells (50), enabling the immune system to play an immune surveillance role (47–49) (**Figure 1**). Compared with adjuvant immunotherapy, neoadjuvant immunotherapy seems to be more advantageous (47–49). In 2016, researchers validated this idea in mouse models of spontaneously metastatic breast cancer where neoadjuvant therapy was superior to adjuvant immunotherapy in eradicating distant micrometastases (51). In human studies, neoadjuvant immunotherapy has been explored in a variety of tumors, such as lung cancer (18, 52), melanoma (20–23), and glioblastoma (26, 27).

### Synergistic effect with radiotherapy

**Figure 2:**
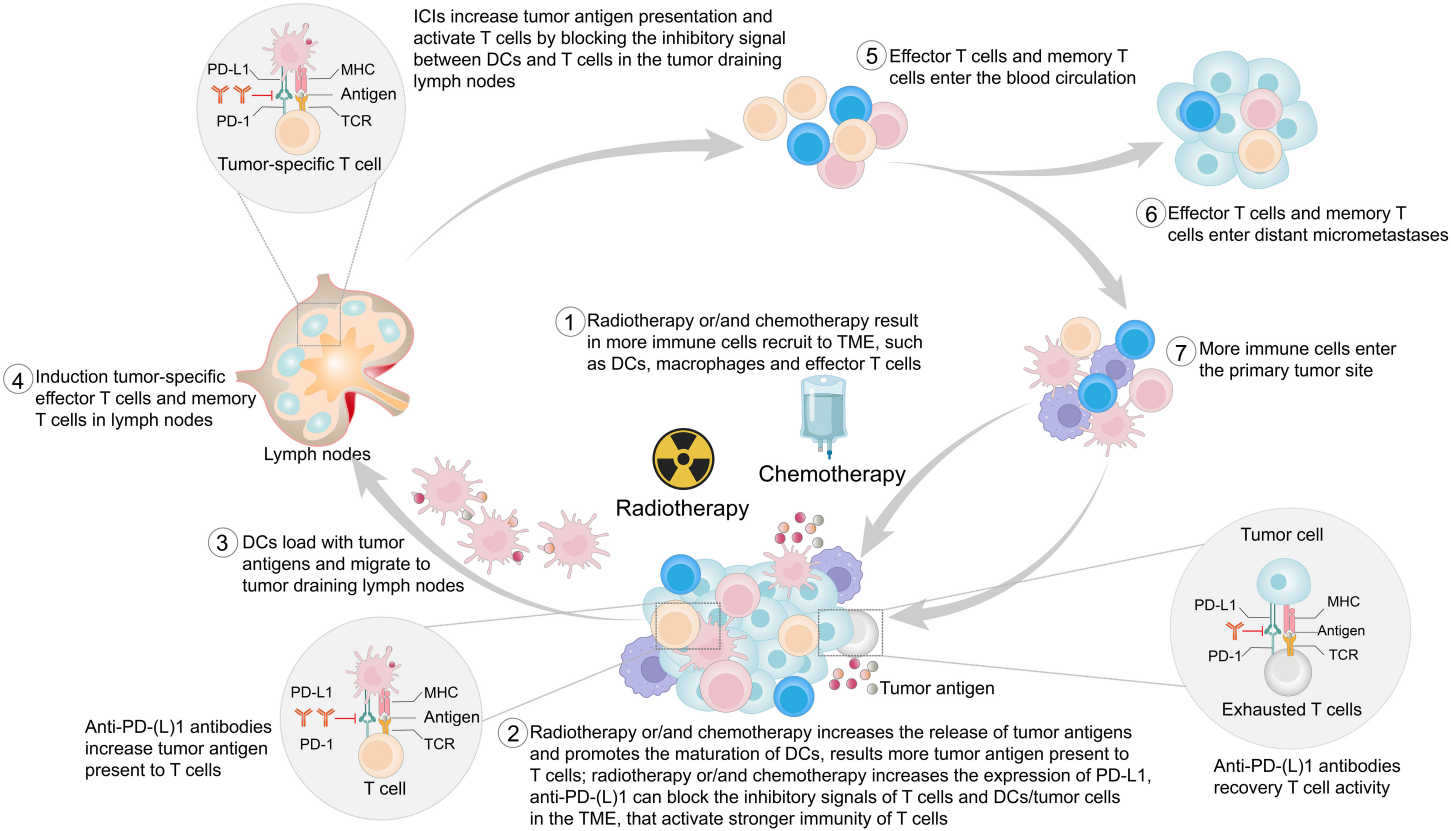
This figure provides a stepwise overview of the potential mechanism of the synergistic antitumor effect of immune checkpoint inhibitors (ICIs) combined with radiotherapy and/or chemotherapy. PD-1, programmed cell death protein 1; PD-L1, programmed cell death 1 ligand 1; MHC, major histocompatibility complex; TCR, T-cell receptor; TME, tumor microenvironment; DCs, dendritic cells.

In addition to local effects, radiotherapy sometimes leads to tumor regression in unirradiated lesions, a phenomenon known as the abscopal effect (53–55). Demaria et al. (56) first attributed the abscopal effect to immune-mediated mechanisms, and others also confirmed that radiotherapy could activate the body’s immune system (57, 58). ICIs block the inhibitory signals between immune cells and tumor cells, increasing the presentation of tumor antigens (47–49). Radiotherapy also modulates the immune system in multiple ways (**Figure 2**). Radiotherapy induces immunogenic cell death, upregulates chemokines or cytokines, and recruits immune cells to the TME (59–61). Radiotherapy activates the type I interferon response *via* the stimulator of interferon genes pathway. Type I interferon is a well-known mediator of DC recruitment and maturation (62–64). Importantly, radiation therapy serves as an *in situ* vaccine by increasing the release of tumor antigens and the uptake of antigens by DCs (65–67). Last but not least, radiotherapy increases the expression of PD-L1 (59, 68). Although the interactions between ICIs and radiotherapy are not well established, their combination enhances the antitumor effects (69–73), which has been confirmed in preclinical models (59, 73, 74). In patients with EC, this combination is now being actively considered as a first-line treatment (75, 76).

### Synergistic effects with chemotherapy

Chemotherapy has dual modulatory effects on the immune system. In addition to its well-known immunosuppressive effects, chemotherapy has recently been found to have immune-activating properties (77, 78). Chemotherapy promotes immunogenic cell death and initiates antitumor immune responses (79, 80). Chemotherapy suppresses immunosuppressive cells, activates effector cells, and increases DC and T-cell infiltration (80–86). Chemotherapy kills tumor cells, which releases tumor antigens (87). Both preclinical and clinical studies found that commonly used chemotherapeutic agents, such as oxaliplatin, cisplatin, paclitaxel, and 5-fluorouracil, promote the upregulation of PD-L1 expression in EC and other cancers (86, 88–94). Therefore, chemotherapy is also synergistic with ICIs (**Figure 2**). In advanced EC, compared with chemotherapy alone, the combination of chemotherapy and ICIs shows clinical and statistical survival benefits (9–17), and this combination has been approved for the treatment of a variety of tumors (82).

In summary, ICIs exert antitumor effects by modulating the body’s immune system instead of killing tumor cells directly. In accumulating studies, durable tumor control was achieved with better effects than traditional chemotherapy and/or radiotherapy (95–98). This unique mechanism provided the rationale for neoadjuvant immunotherapy, whereby long-term survival is expected. It is theoretically feasible to combine chemotherapy and/or radiotherapy with ICIs for neoadjuvant treatment of locally advanced resectable EC.

## Clinical studies

### Reported clinical studies

**Figure 3:**
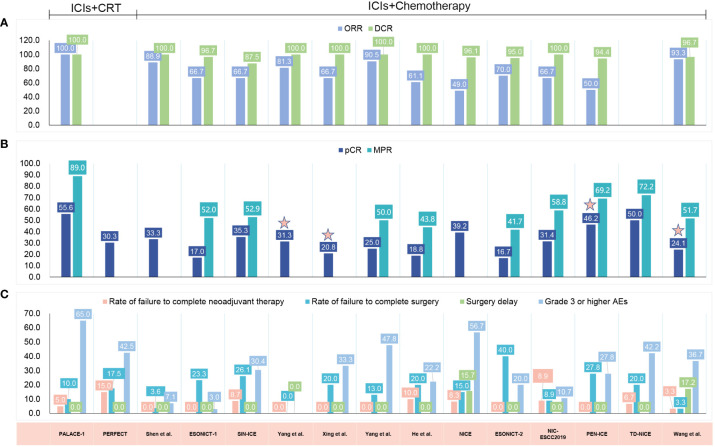
Published clinical studies on immune checkpoint inhibitor (ICI) neoadjuvant therapy in resectable esophageal cancer (EC). **(A)** The radiologic response. **(B)** The pathological response. **(C)** The safety results. ICIs, immune checkpoint inhibitors; CRT, chemoradiotherapy; ORR, objective response rate; DCR, disease control rate; pCR, pathologic complete response; MPR, major pathological response; AEs, adverse events; ESCC, esophageal squamous cell carcinoma; EAC, esophageal adenocarcinoma; NA, not available.

Multiple clinical trials have explored the efficacy and safety of immunotherapy against resectable EC in the neoadjuvant setting (**Table 1**, **Figure 3**). Initially, clinical trials examined neoadjuvant immunotherapy plus chemoradiotherapy (CRT) (99, 100), and recent trials have evaluated neoadjuvant chemoimmunotherapy (101–112) and neoadjuvant immunotherapy plus antiangiogenic therapy (113). Current reported clinical trials on neoadjuvant immunotherapies are mainly single-arm studies with small samples. Most of them were conducted in China and were directed against ESCC.

**Table 1:** Reported clinical trials of neoadjuvant immunotherapy for the treatment of resectable esophageal cancer.

	PALACE-1	PERFECT	Shen et al.	ESONICT-1	SIN-ICE	Yang et al.	Xing et al.	Yang et al.	He et al.	NICE	ESONICT-2	NIC-ESCC2019	PEN-ICE	TD-NICE	Wang et al.
Study phase	Ib	II	II	II	Pilot study	Pilot study	II	Pilot study	II	II	II	II	II	II	Ib
Enrolled patients	20	40	28	30	23	16	30	23	20	60	20	56	18	45	30
Pathological type	ESCC	EAC	ESCC	ESCC	ESCC	ESCC	ESCC	ESCC	ESCC	ESCC	ESCC	ESCC	ESCC	ESCC	ESCC
Clinical stage	II-IVA	II-IVA	II-IVA	III-IV	II-IVA	II-IVA	II-IVA	II–III	III-IVa	III-IVA	III-IVA	II-IVA	II–IVA	II-IVA	II-III
Immune drugs	Pembrolizumab	Atezolizumab	Nivolumab, pembrolizumab, camrelizumab	sintilima	sintilimab	Camrelizumab	Toripalimab	Camrelizumab	Toripalimab	Camrelizumab	Toripalimab	Camrelizumab	Pembrolizumab	Tislelizumab	Camrelizumab
Immune targets	PD-1	PD-L1	PD-1	PD-1	PD-1	PD-1	PD-1	PD-1	PD-1	PD-1	PD-1	PD-1	PD-1	PD-1	PD-1
Chemotherapeutic drugs	carboplatin, paclitaxel	carboplatin, paclitaxel	nab-paclitaxel, carboplatin	albumin-bound paclitaxel, cisplati	Docetaxel/albumin-bound paclitaxel, nedaplatin	Paclitaxel,carboplatin	Paclitaxe, cisplatin	nab-paclitaxel, carboplatin	Paclitaxel, carboplatin	nab-paclitaxel, carboplatin	Docetaxel, cisplatin	nab-paclitaxel, cisplatin	Platinum-based two-drug	nab-paclitaxel, carboplatin	nab-paclitaxel, nedaplatin, apatinib
Chemotherapy cycle	5, Q1W	5, Q1W	2, Q3W	2, Q3W	3, Q3W	2, Q3W	2, Q3W	2, Q3W	2, Q3W	2, Q3W	2, Q3W	2, Q3W	3, Q3W	3, Q3W	2-4, Q3W
Radiotherapy	23 fractions of 1.8 Gy	23 fractions of 1.8 Gy	NA	NA	NA	NA	NA	NA	NA	NA	NA	NA	NA	NA	NA
Time from neoadjuvant therapy to surgery	4-6 weeks	1-3 weeks	3-5 weeks	4-6 weeks	4-6 weeks	4 weeks	4-6 weeks	3-6 weeks	4-6 weeks	4-6 weeks	4-6 weeks	6 weeks	4-6 weeks	4-6 weeks	4-8 weeks
Primary endpoints	Safety	Feasibility	Safety, feasibility	pCR, AEs	pCR, safety	pCR	pCR	Safety, feasibility	Safety, feasibility, MPR	pCR	pCR, AEs	pCR	Safety, efficacy	MPR	Safety

ESCC, Esophageal squamous cell carcinoma; EC, Esophagus adenocarcinoma; NA, Not Applicable; pCR, Pathologic complete response; MPR, Major pathological response; AE, Adverse events.

### Efficacy

The efficacy outcomes are graphically summarized in **Figure 3**. Among the 15 included studies, 13 evaluated the radiologic response with the Response Evaluation Criteria in Solid Tumors (RECIST) (99, 101–111), with the ORR fluctuating from 49.0% to 100% and the disease control rate (DCR) fluctuating from 87.5% to 100% (**Figure 3**). All of these studies reported R0 resection rates ranging from 80.5% to 100.0%.

The pCR rate was reported by all studies, and 10 of 15 reported the major pathological response (MPR) rate. Five studies did not report MPR (100, 101, 104, 105, 108). The addition of ICI to CRT led to pCR rates of 55.6% and 30.3%, respectively (99, 100), and led to an MPR rate of 89.0% (99) (**Figure 3**). When neoadjuvant ICI was combined with chemotherapy, different pCR and MPR rates were achieved, with the pCR ranging from 16.7% to 50.0% (101–112) and the MPR from 41.7% to 72.2% (102, 103, 106, 107, 109–112) (**Figure 3**). Combining chemotherapy with camrelizumab and apatinib led to a pCR rate of 24.1% and an MPR rate of 51.7% (113) (**Figure 3**). It is noteworthy that 11 of these 15 studies noted that a pCR was defined as the absence of residual tumor in both the primary tumor and lymph nodes (ypT0N0) (99–103, 106–110, 112), whereas the other 4 studies did not explicitly indicate ypT0N0 was required for a pCR (104, 105, 111, 113).

When compared with the classic CROSS (49%) (114) or NEOCRTEC5010 study (43.2%) (4), ICIs combined with chemotherapy showed no significant advantage in the pCR rate. In studies where ICIs were combined with CRT, such as the PALACE-1 study (99), a better pCR of 55.6% was reported. In another PERFECT study (100), a higher pCR in patients with EAC was also reported (30.3% vs. 23% for CRT). Notably, the results of these small-scale preliminary studies were unreliable, and additional large-scale studies are needed to confirm the efficacy of neoadjuvant immunotherapy in patients with locally advanced resectable EC.

### Safety

The safety results are graphically summarized in **Figure 3**. The rates of failure to complete neoadjuvant therapy varied from 0% to 15.0%, mainly due to treatment-related AEs (TRAEs) (99, 100, 108), patient decisions (107, 110, 112) or disease progression (100). One study did not report the specific reason for 2 patients not proceeding to the planned neoadjuvant treatment (103). The rates of failure to undergo resection ranged from 0% to 40%. There were various reasons reported for not proceeding to resection: disease progression (99, 100, 102, 103, 105, 108, 111, 112), patient refusal (100–103, 105–112), death (99, 100, 108), TRAEs (105), compromised general condition (110) and dropped out (108). Notably, in the ESONICT-2 study, 8 of 20 patients failed to undergo surgery, 3 patients refused surgery due to symptom relief, and another 5 patients were not suitable for radical surgery, but no specific reasons were reported (109). Surgical delays were reported in 2 of the 15 included studies, and all were attributed to TRAEs (108). The rates of patients experiencing surgical delay were 15.7% (108) and 17.2% (113), respectively.

In the two studies that added ICI to CRT, the incidence of grade 3 and higher AEs was 65.0% and 42.5%, respectively (99, 100). Most of these AEs were lymphopenia or gastrointestinal related (i.e., anorexia or nausea) and occurred during the neoadjuvant treatment period (99, 100). During neoadjuvant treatment with ICI chemotherapy, reported rates of AEs ranged from 3.0% to 56.7%. Here, the most frequently reported AEs were hematological disorders (101–103, 105–112), followed by gastrointestinal-related (i.e., anorexia, vomiting, diarrhea) (103, 107, 110–112), and immune-related AEs (i.e., enteritis, hyperthyroidism, dermatitis) (105, 109, 110). Rash (101, 110), pneumonia (105, 108), alopecia (103, 111), fatigue (107, 111), fever (108) and blurred vision (108) have been reported in only a few studies. One study reported AEs associated with neoadjuvant therapy; however, these events were not reported in a graded manner (104). The combination of chemotherapy with ICI and apatinib led to 36.7% of patients experiencing grade 3 AEs. No grade 4 or 5 AEs were reported (113).

### Registered clinical trials

More clinical trials can be found at ClinicalTrials.gov (**Table 2**). In most of them, either CRT or chemotherapy is adopted in combination with ICI. In others, ICI is used alone (NCT04215471, NCT04196465, NCT03987815, NCT02735239), combined with radiotherapy (NCT05176002, NCT03200691), combined with both multitargeted small molecule inhibitors and CRT or chemotherapy (NCT04929392, NCT04757363, NCT04666090), combined with both CRT and anti-EGFR antibody (NCT05355168), or used in combination with another ICI and CRT (NCT03776487).

**Table 2:** Registered clinical trials in ClinicalTrials.gov investigating neoadjuvant immunotherapy for the treatment of resectable esophageal cancer.

Neoadjuvant treatment protocol	NCT Number	Pathological type	Phase	Intervention	Sample size	Primary endpoint	Status
ICIs+CRT	NCT05357846	ESCC	3	Tislelizumab/Paclitaxel/Cisplatin /Radiation	422	OS	Not yet recruiting
	NCT05323890	ESCC	2	Tislelizumab/ Albumin paclitaxel/Cisplatin/Radiation	15	MPR, pCR	Recruiting
	NCT05043688	ESCC	2	SHR-1210/Albumin paclitaxel/Carboplatin/Radiation	204	pCR	Not yet recruiting
	NCT04974047	ESCC	2	Tislelizumab/Paclitaxel/Cisplatin/Radiation	65	pCR	Recruiting
	NCT04973306	ESCC	2/3	Tislelizumab/Paclitaxel/Carboplatin/Radiation	176	pCR, OS	Recruiting
	NCT04888403	ESCC	2	Toripalimab/Albumin paclitaxel/Nedaplatin/Radiation	45	pCR	Not yet recruiting
	NCT04776590	EC	2	Tislelizumab/Albumin paclitaxel/Caboplatin/Radiation	30	pCR	Recruiting
	NCT04644250	ESCC	2	Toripalimab/Paclitaxel liposome/Carboplatin/Radiation	32	pCR	Recruiting
	NCT04568200	ESCC	2	Durvalumab/Paclitaxel/Carboplatin/Radiation	60	Tumor and pathological response	Recruiting
	NCT04437212	ESCC	2	Toripalimab/Paclitaxel/Cisplatin/Radiation	20	MPR	Recruiting
	NCT04435197	ESCC	2	Pembrolizumab/Carboplatin/Paclitaxel/Radiation	143	pCR	Recruiting
	NCT04177875	EC	2	Teripalimab/Docetaxel or albumin paclitaxel/Cisplatin/Radiation	44	MPR/ORR	Recruiting
	NCT03792347	ESCC	2	Pembrolizumab/Paclitaxel/Carboplatin/Radiation	143	pCR	Recruiting
	NCT03544736	EC	1/2	Nivolumab/Paclitaxel/Carboplatin/Radiation	30	TEAE	Recruiting
	NCT03490292	EC/GEC	1/2	Avelumab/Paclitaxel/Carboplatin/Radiation	24	DLTs/pCR	Recruiting
	NCT03064490	EGC	2	Pembrolizumab/Paclitaxel/Carboplatin/Radiation	38	pCR	Recruiting
	NCT03044613	EC/GC/EGC	1	Nivolumab/Relatlimab/Carboplatin/Paclitaxel/Radiation	25	TRAE	Recruiting
	NCT02844075	ESCC	2	Pembrolizumab/Taxol/Carboplatin/Radiation	18	pCR	Active, not recruiting
ICIs+Chemo	NCT05476380	ESCC	2	Camrelizumab/Paclitaxel/Cisplatin	39	pCR	Recruiting
	NCT05302011	ESCC	2	Pembrolizumab/Docetaxel/Carboplatin or Cisplatin	30	Tumor and pathological response	Recruiting
	NCT05281003	ESCC	2	Pembrolizumab/Paclitaxel/Cisplatin	128	pCR	Not yet recruiting
	NCT05244798	ESCC	3	Tislelizumab/Albumin paclitaxel/Cisplatin Tislelizumab/Albumin paclitaxel/Cisplatin/Radiation	360	pCR	Not yet recruiting
	NCT05213312	ESCC	2/3	Nivolumab/Paclitaxel or 5Fluorouracil/Cisplatin	90	pCR	Recruiting
	NCT05189730	ESCC	2	Tislelizumab/Paclitaxel/Cisplatin	80	pCR, AEs	Recruiting
	NCT05182944	ESCC	2	Camrelizumab/Albumin paclitaxel/Cisplatin	130	pCR, DFS	Recruiting
	NCT05174325	ESCC	2	Sintilimab/Albumin paclitaxel/Cisplatin	30	pCR	Recruiting
	NCT05050760	ESCC	NA	Camrelizumab/Oxaliplatin/Docetaxel/Tegafur	55	Safety, Feasibility	Not yet recruiting
	NCT05028231	ESCC	NA	PD-1 or PD-L1/Chemotherapy	46	pCR	Recruiting
	NCT04937673	ESCC	2	Camrelizumab/Albumin paclitaxel or paclitaxel/Cisplatin	40	Biomarkers related to pCR	Not yet recruiting
	NCT04848753	ESCC	3	Toripalimab/Paclitaxel/Cisplatin	500	EFS	Recruiting
	NCT04844385	ESCC	2	Toripalimab/Albumin paclitaxel/Nedaplatin	83	2-year PFS rate	Recruiting
	NCT04813523	GEJAC	2	Pembrolizumab/5Fluorouracil /Cisplatin/	30	MPR	Recruiting
	NCT04807673	ESCC	3	Pembrolizumab/Paclitaxel/Cisplatin	342	EFS	Recruiting
	NCT04804696	ESCC	2	Toripalimab/Paclitaxel/Cisplatin	53	pCR	Recruiting
	NCT04767295	ESCC	2	Camrelizumab/Albumin paclitaxel/Carboplatin	28	ORR	Recruiting
	NCT04625543	ESCC	2	Sintilimab/Paclitaxel/Cisplatin	100	MPR	Not yet recruiting
	NCT04506138	ESCC	1/2	Camrelizumab/Albumin paclitaxel/Carboplatin	46	pCR/MRP	Recruiting
	NCT04460066	EC	1/2	Anti-PD-L1 antibody/Albumin paclitaxel/Cisplatin	70	MPR	Not yet recruiting
	NCT04389177	ESCC	2	Pembrolizumab/Carboplatin/Paclitaxel	50	MPR	Recruiting
	NCT04280822	EC	3	JS001/Paclitaxel/Cisplatin	400	3 years EFS/5 years EFS	Recruiting
	NCT04221555	GAC/GEJAC	2	Durvalumab/Docetaxel/Oxaliplatin/S-1	68	pCR	Recruiting
	NCT04006041	ESCC	2	Toripalimab/Paclitaxel/Cisplatin	44	pCR	Recruiting
	NCT03946969	ESCC	1/2	Sintilimab/Liposomal paclitaxel/Cisplatin/S-1	40	TEAE	Recruiting
	NCT03917966	ESCC	2	SHR-1210/Docetaxel/Nedaplatin	40	ORR/MPR	Recruiting
	NCT03914443	ESCC	1	Nivolumab/5Fluorouracil /Cisplatin/Docetaxel	36	DLTs	Active, not recruiting
	NCT03448835	GC/GEJC	2	Atezolizumab/Capecitabine/Oxaliplatin/Docetaxel	20	AE	Recruiting
ICIs alone	NCT04215471	ESCC	2	Anti-PD-L1 antibody SHR-1316	30	OR	Not yet recruiting
	NCT04196465	EC/GC/Liver Cancer	2	Anti-PD-L1 antibody IMC-001	48	MPR	Recruiting
	NCT03987815	ESCC	2	Nivolumab	20	MPR	Recruiting
	NCT02735239	EC	1/2	Durvalumab	75	AE/DLT	Active, not recruiting
ICIs+Radiation	NCT05176002	ESCC	1/2	Camrelizumab/Radiation	26	MPR, AEs	Recruiting
	NCT03200691	ESCC	2	Anti-PD-1 antibody SHR-1210/Radiation	21	pCR	Unknown status
ICIs+CRT+Multi-targeted inhibitor	NCT04929392	EC/GEC	2	Pembrolizumab/Paclitaxel/Carboplatin/Radiation/Lenvatinib Mesylate	24	pCR, cCR	Recruiting
ICIs+Chemo+ Multi-targeted inhibitor	NCT04757363	EGC	2	Nivolumab/Regorafenib/Oxaliplatin/Leucovorin/ 5-FU	35	6-month PFS	Recruiting
	NCT04666090	ESCC	2	Carillizumab/Albumin paclitaxel/Nedaplatin/Apatinib	38	MPR	Recruiting
ICIs+CRT+anti-EGFR antibody	NCT05355168	ESCC	1/2	Camrelizumab/Nimotuzumab/Chemoradiotherapy	57	pCR, MPR	Recruiting
Dual ICIs+CRT	NCT03776487	GC/GAC/GEJAC	1/2	Ipilimumab/Nivolumab/5Fluorouracil/Oxaliplatin/Radiation	30	AE	Recruiting

AE, Adverse events; cCR, clinical complete response; DLT, Dose limiting toxicity; EC, Esophagus cancer; EGC, Esophagogastric cancer; EGFR, Epidermal growth factor receptor; EFS, Event free survival; ESCC, Esophageal squamous cell carcinoma; GAC, Gastric adenocarcinoma; GC, Gastric cancer; GEJAC, Gastroesophageal junction adenocarcinoma; MPR, Major pathological response; NA, Not Applicable; OR, Objective response; ORR, Objective remission rate; pCR, Pathologic complete response; PFS, Progression free survival; TEAE, Treatment emergent adverse events; TRAE, Treatment-related adverse events.

In addition to these phase 2 studies, several phase 3 studies deserve special attention. Hong et al. designed a randomized controlled trial (RCT) to compare PD-1 inhibitors combined with preoperative CRT versus neoadjuvant CRT for locally advanced ESCC (NCT05357846). The KEYSTONE-002 study was designed to evaluate the efficacy and safety of pembrolizumab in combination with chemotherapy for preoperative neoadjuvant therapy and then the continued use of pembrolizumab as adjuvant therapy postoperatively compared with neoadjuvant CRT and surgery for locally advanced ESCC (NCT04807673). Two other studies are comparing the efficacy of neoadjuvant chemotherapy combined with immunotherapy versus neoadjuvant chemotherapy in resectable ESCC (NCT04848753, NCT04280822). Immunotherapy plus neoadjuvant chemotherapy versus immunotherapy plus neoadjuvant CRT is also being studied (NCT05244798).

In summary, neoadjuvant chemoradiotherapy remains the standard treatment for locally advanced esophageal cancer, and neoadjuvant immunotherapy is in the clinical trial stage. No indications for neoadjuvant immunotherapy are currently authorized.

## The challenges

**Figure 4:**
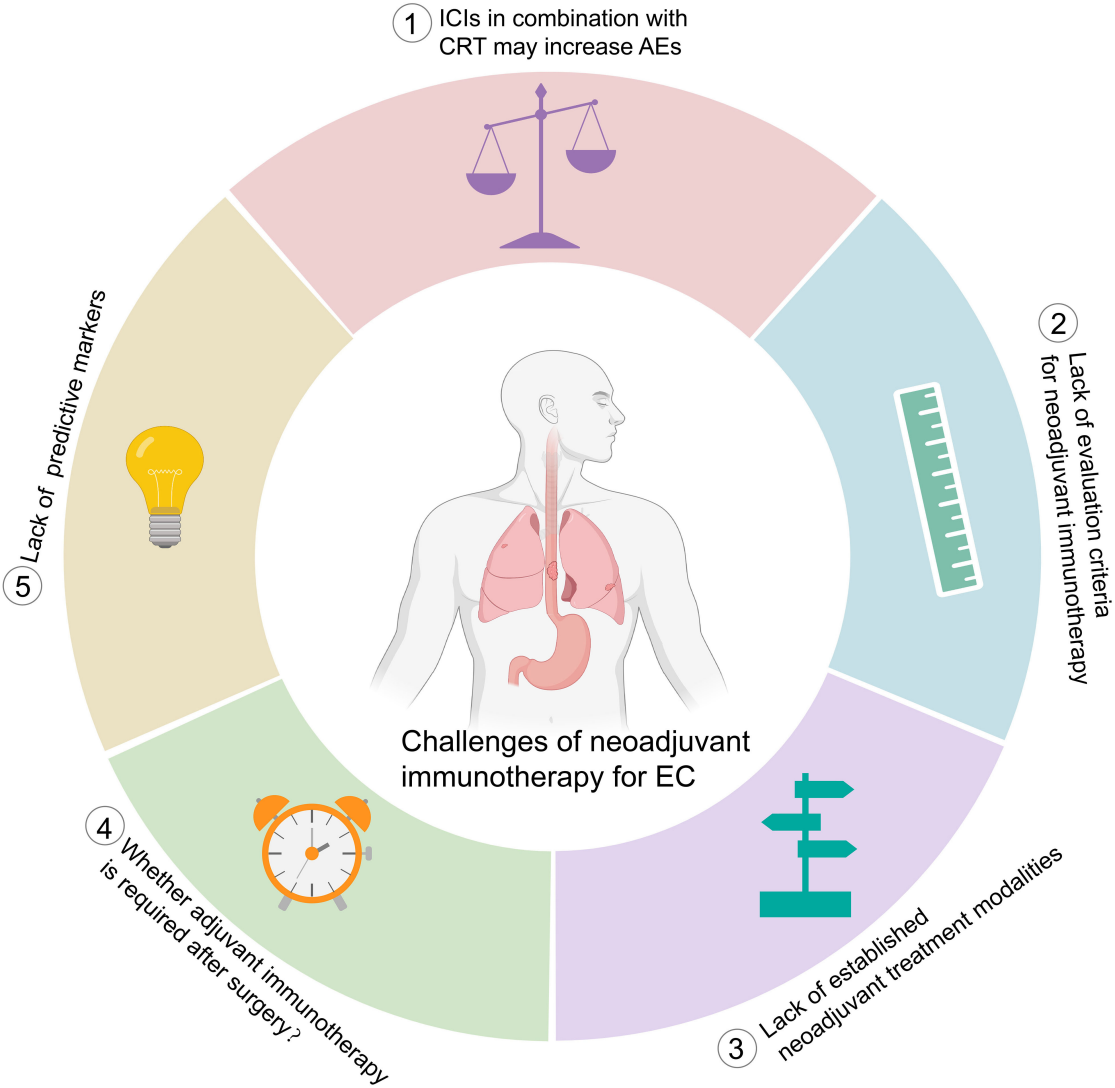
Challenges of neoadjuvant immunotherapy for esophageal cancer. ICIs, immune checkpoint inhibitors; CRT, chemoradiotherapy, AEs, adverse events; EC, esophageal cancer.

Neoadjuvant immunotherapy in EC is still in its infancy, and many unanswered questions remain. Here, we summarize the challenges and future directions (**Figure 4**).

### AEs

ICIs might cause specific toxicity profiles, i.e., immune-related AEs, different from those of chemo- or radiotherapy (70, 115). In addition, when combination therapy is adopted, the type and severity of TRAEs might be more complex (70, 116). The PACIFIC study reported a higher incidence of treatment discontinuation due to AEs in the ICI plus CRT group than in the CRT alone group (15.4% vs. 9.8%) (117). In a meta-analysis including 3,144 patients, ICIs plus chemotherapy had a significantly higher incidence of AEs in non-small cell lung cancer (NSCLC) (118). Similarly, the CheckMate 648 study reported that patients with advanced ESCC treated with nivolumab plus chemotherapy had a higher incidence of grade 3-4 TRAEs than those treated with chemotherapy alone (47% vs. 36%) (14).

In neoadjuvant immunotherapy for EC, a combination of CRT with ICIs increased the pCR rate, but the incidence of grade 3 or worse AEs was high, and deaths during treatment were reported (99, 119). In the PALACE-1 study, one patient died of esophageal hemorrhage while awaiting surgery (99). In another phase II clinical study (NCT02844075), among the 26 patients who underwent surgery after treatment with pembrolizumab and CRT, 2 patients died of acute lung injury after surgery (119). In radiation-free therapies, although the grade 3-4 AE rate was decreased (120), treatment-related surgical delay was reported (108). In the NICE study, surgery was delayed by a median of 19 days due to AEs (108).

The current available toxicity data were all collected from single-arm studies with limited numbers of patients. Large randomized controlled studies are warranted to establish the safety of ICI neoadjuvant treatment of EC. From the above reports, lung injury is a concern when CRT and ICIs are used concurrently. In clinical practice, the extent of cancer lesions or lymph node metastases and the dose of radiation delivered to the lungs should be clearly defined for patients receiving CRT in combination with ICIs. In addition, delayed toxicities remain elusive due to insufficient follow-up.

### Response evaluation

Pathologic response is the most common surrogate endpoint for relapse-free survival and OS in cancer neoadjuvant therapy (48). pCR is defined as the absence of any viable tumor in the surgically resected specimens and all sampled lymph nodes (121). MPR, described as ≤10% of residual viable tumor (RVT) in a surgically resected specimen, has been proposed as an alternative parameter (122). To date, pCR and MPR are the most commonly used metrics for assessing the response to neoadjuvant immunotherapy. However, other criteria for pathological assessment have been used for EC. In the PERFECT study, the pathologic response was assessed according to Mandard’s tumor regression grade score (100).

It is highly appreciated when the pathological response is reported in a uniform and reproducible manner to allow for valid cross-study comparisons. However, the pathological response criteria that were developed for chemotherapy and/or radiotherapy may not be suitable for neoadjuvant immunotherapy. In addition, OS was reported to be correlated with the response spectrum of RVT, implying that if assessments beyond pCR and MPR could be performed, prognostication could potentially be available for all patients (123, 124).

Recently, immune-related pathologic response criteria (irPRC) have been developed, that is, scoring 0 to 100% irRVT in 10% intervals (125). This approach, first described for neoadjuvant anti-PD-1 monotherapy in NSCLC (18), has been extended to other tumor types and combination treatment regimens (126). %irRVT =viable tumor area/total tumor bed area, whereby the total tumor bed=regression bed +RVT+ necrosis. The regression bed is defined as the area of immune-mediated tumor clearance characterized by tumor-infiltrating immune cells, tumor cell death with cholesterol clefts, and hallmarks of tissue repair, such as neovascularization and proliferative fibrosis (125). Currently, irPRC has not been adopted in EC, and more studies are needed to confirm the prognostic value of %irRVT.

Additionally, neoadjuvant immunotherapy may bring difficulties to radiological response evaluation since the tumor regression pattern seems different from what may happen during chemo- or radiotherapy. Radiographic responses such as pseudoprogression or a delayed response to immunotherapy have been frequently reported (127–129) and are expected during the neoadjuvant immunotherapy of EC. However, no such observations were reported in the 15 included studies. None of the studies reported any mismatch between the radiological and pathological responses. Whether this was due to the limited number of patients is unknown. We predict that as more studies related to neoadjuvant immunotherapy become available, such discrepancies in the radiological response between immunotherapy and chemotherapy and/or radiotherapy will be revealed. This will pose a challenge in the near future.

### Treatment modalities

In neoadjuvant immune combination therapy, the chemotherapy regimens were mainly paclitaxel and platinum (99, 100, 111, 112) (**Figure 3**). Chemotherapy was administered weekly for 5 weeks in neoadjuvant therapy with CRT and ICIs (99, 100, 119, 130, 131). In neoadjuvant therapy using ICIs and chemotherapy, preoperative treatment was generally administered every 3 weeks for 2 cycles (101–110). However, a higher pCR and MPR were achieved in two studies that used 3 cycles of treatment (111, 112).

Theoretically, chemotherapy may induce lymphopenia and selectively deplete immunosuppressive cells (80), while ICI therapy may result in the proliferation of tumor-specific T cells (48). Therefore, ICI therapy applied after chemotherapy may allow for the proliferation of effector T cells and reduce the possibility of killing tumor-specific T cells with the chemotherapeutic drugs, producing better antitumor efficacy. In a retrospective study, ICIs used 1-10 days after chemotherapy was superior to ICIs used before or concurrent with chemotherapy in patients with refractory lung cancer (132). In addition, Xing et al. (105) showed that in neoadjuvant treatment of EC, sequential immunotherapy after chemotherapy was more effective than concurrent chemo-immunotherapy.

From these reported results, ICI combined with CRT achieved higher rates of pCR and MPR over chemotherapy (120). It should be kept in mind that the results from these small-scale preliminary studies are unstable and inconclusive. Whether pCR from variant treatment modalities could be translated into improved survival remains largely unknown. The most suitable treatment modality for neoadjuvant therapy has yet to be determined. It was interesting to see other treatment modalities such as ICI in combination with radiotherapy (NCT05176002, NCT03200691), kinase inhibitors (NCT04929392, NCT04757363, NCT04666090), or ICIs alone (NCT04215471, NCT04196465, NCT03987815, NCT02735239) are being evaluated in different trials, in addition to the mainstream CRT or chemotherapy combination (**Table 1**).

### Adjuvant immunotherapy

In the NADIM study, patients with resectable stage IIIA NSCLC received neoadjuvant treatment with platinum-based chemotherapy plus nivolumab before surgical resection, followed by adjuvant nivolumab monotherapy for 1 year. This study showed that the treatment regimen was well tolerated, and at 24 months, the PFS was 77% and the OS was 90% (19). Based on the results of the NADIM study, several ongoing phase III clinical studies of lung cancer (KEYNOTE 617, IMPOWER 030, AEGEAN) or breast cancer (KEYNOTE-522) are evaluating patients receiving ICIs neoadjuvant therapy followed by 1 year of ICIs after surgery. Similar studies are underway in the EC (NCT05213312, NCT05189730, NCT05182944, NCT04813523, NCT02844075, KEYSTONE-002). In a phase II clinical study (NCT02844075), ESCC patients received neoadjuvant immunotherapy, followed by surgery and immunotherapy for 2 years. The preliminary results of this study showed that at a median follow-up of 11.7 months, the median OS was not reached and the 6- and 12-month OS rates were 89.3% and 82.1%, respectively (119).

Theoretically, postoperative adjuvant ICI therapy is a reasonable option to prevent postoperative recurrence and metastasis. However, there are some issues that deserve our attention. As one example, in patients with HER2-positive early-stage breast cancer, neoadjuvant anti-HER2 therapy plus chemotherapy followed by surgery and adjuvant therapy with anti-HER2 therapy was beneficial only for patients without a pCR (133). These results prompted us to think that adjuvant therapy could be less relevant in selected populations, such as patients with a pCR (134). Among the ongoing clinical studies of neoadjuvant immunotherapy for EC, two studies are applying adjuvant treatment only for patients who have not achieved a pCR (NCT05213312, NCT05189730); in the KEYSTONE-002 study and the NCT04813523 study, postoperative adjuvant therapy is being administered to all patients; and in the NCT05182944 study, different adjuvant therapy is being used for patients with pCR and non-PCR. It remains unknown whether all patients should receive ICIs after surgery.

Additionally, prolonged use of ICIs may lead to increased AEs. In mouse tumor models, compared with mice given 2 doses of neoadjuvant immunotherapy, mice treated with 2 doses of neoadjuvant immunotherapy plus 4 adjuvant immunotherapy did not display any significant increase in OS but they did have an increase in immune-related AEs (135). Furthermore, the optimal treatment interval and duration of adjuvant ICIs related to treatment compliance and financial toxicity also represent significant challenges (134). The duration of adjuvant therapy in current clinical studies is very inconsistent. Of note, the half-life of most anti-PD-1 antibodies is 12-20 days regardless of the dose (136), suggesting a longer interval between adjuvant anti-PD1 doses might be optimal. All in all, the development of a postoperative adjuvant treatment strategy must be based on a comprehensive assessment of the survival benefit, treatment compliance, and the toxicities and side effects.

### Predictive markers

PD-L1 expression could be used to predict the efficacy of pembrolizumab in advanced EC (9). However, for neoadjuvant therapy, the current data do not support PD-L1 expression as a biomarker in EC (99, 100, 103, 105–108, 110, 111, 113). Theoretically, the level of CD8+ T infiltration into the TME correlates with the efficacy of immunotherapy, since blocking the PD-1/PD-L1 interaction can restore the tumor-killing effect of exhausted CD8+ T cells (137). However, in neoadjuvant immunotherapy, recent studies showed no significant difference in CD8+ T cells between responders and nonresponders (99, 100, 106, 107). Recently, TCF-1+ CD8+ T cells were found to be precursor exhausted CD8+ T cells with stem cell-like properties, and TCF-1+CD8+ T cells were associated with immunotherapy efficacy (138, 139). The PALACE-1 study revealed that compared with nonpCR patients, there was an increased percentage of TCF-1+ cells in the samples from pCR patients (99). These findings are consistent with recent reports (139–141).

Genomic analysis showed that in some studies, TMB was higher in the pCR group compared to the nonpCR group (106, 108). However, He et al. (107) indicated TMB failed to distinguish the two groups. Beyond TMB, immune-related genes have received increasing attention. The PERFECT study found that those who responded to neoadjuvant immunotherapy had a significantly higher IFN-γ score at baseline, while those who did not respond to neoadjuvant immunotherapy had higher expression of ICI resistance-related genes in their tumor tissues despite the presence of cytotoxic T-lymphocyte infiltration (100). He et al. (107) also found that responders had higher chemokine CXCL5 expression and lower chemokine CCL19 and UMODL1 expression compared with nonresponders.

In summary, TCF-1+ CD8+ T cells, TMB and immune-related genes deserve further exploration in larger-scale clinical studies for predicting the response to neoadjuvant immunotherapy for EC. Going forward, the identification of biomarkers reflecting complex tumor-immune system interactions and immune system-host interactions will help us to identify patients who will truly benefit from neoadjuvant immunotherapy. In addition, although traditional imaging techniques cannot accurately reflect the pathological changes of tumor tissue during neoadjuvant therapy, with advances in imaging technology, particularly positron emission tomography technology, we may be able to label specific immune cells, checkpoint molecules, or markers of metabolic processes associated with the neoadjuvant treatment response or resistance to guide or adjust clinical decision-making (142, 143).

## Conclusion

Although the use of immunotherapy for preoperative neoadjuvant therapy versus adjuvant therapy may be theoretically more effective, and neoadjuvant immunotherapy has shown preliminary positive results in resectable EC in some clinical studies, further validation of the feasibility, safety, and efficacy of neoadjuvant immunotherapy in large randomized clinical studies is still needed. In addition, a number of unresolved issues must be addressed before neoadjuvant ICIs strategies can be widely adopted as the standard of care. Identifying predictive biomarkers will be key to selecting appropriate populations, and the role of adding adjuvant therapy must be fully understood. Furthermore, long-term follow-up is needed to determine the long-term outcomes and assess any delayed toxicity. We are confident that neoadjuvant immunotherapy will move forward into a new chapter.
