# Supplementary material for: Neoadjuvant immunotherapy for resectable esophageal cancer: A review
Source: Front Immunol. 2022 Dec 8;13:1051841. doi: 10.3389/fimmu.2022.1051841 (PMC9773255; doi:10.3389/fimmu.2022.1051841)
Supplement: Supplementary file 1 [file Table_1.docx]

Supplementary Table 1. Abbreviations used in the manuscript.

| Abbreviation | Full name |
| --- | --- |
| EC | esophageal cancer |
| nCRT | neoadjuvant chemoradiotherapy |
| pCR | pathological complete response |
| ICIs | immune checkpoint inhibitors |
| ESCC | esophageal squamous cell carcinoma |
| OS | overall survival |
| ORR | objective response rate |
| AEs | adverse events |
| PD-1 | programmed death 1 |
| PFS | progression free survival |
| TMB | tumor mutational burden |
| PD-L1 | programmed cell death-ligand 1 |
| TME | tumor microenvironment |
| DCs | dendritic cells |
| CRT | chemoradiotherapy |
| RECIST | Response Evaluation Criteria in Solid Tumors |
| DCR | disease control rate |
| MPR | major pathological response |
| TRAEs | treatment-related AEs |
| RCT | randomized controlled trial |
| irAEs | immune-related adverse events |
| NSCLC | non-small cell lung cancer |
| RVT | residual viable tumor |
| irPRC | immune-related pathologic response criteria |
